# Supplementary material for: Improving the Stability of Oil Body Emulsions from Diverse Plant Seeds Using Sodium Alginate
Source: Molecules. 2019 Oct 25;24(21):3856. doi: 10.3390/molecules24213856 (PMC6864775; doi:10.3390/molecules24213856)
Supplement: Supplementary file 1 [file molecules-24-03856-s001.pdf]

Supplementary Materials

Improvement of stability of oil body emulsions from diverse plant seeds by sodium alginate

Yuemei Zhang[1], Nan Yang[1, 2, *], Yao Xu[1], Qian Wang[1], Ping Huang[1], Katsuyoshi Nishinari[1, 2] and Yapeng Fang [1, 2]

[1] Glyn O. Phillips Hydrocolloid Research Centre, National "111" Center for Cellular Regulation and Molecular Pharmaceutics, Key Laboratory of Fermentation Engineering (Ministry of Education), Department of Bioengineering and Food Science, Hubei University of Technology, Wuhan 430068, China

[2] Food Hydrocolloid International Science and Technology Cooperation Base of Hubei Province, Hubei University of Technology, Wuhan 430068, China

[*] Correspondence: nanyang@hbut.edu.cn; Tel.: +86 (0) 27-88015996

Received: 23 August 2019; Accepted: 25 October 2019; Published: 25 October 2019

a

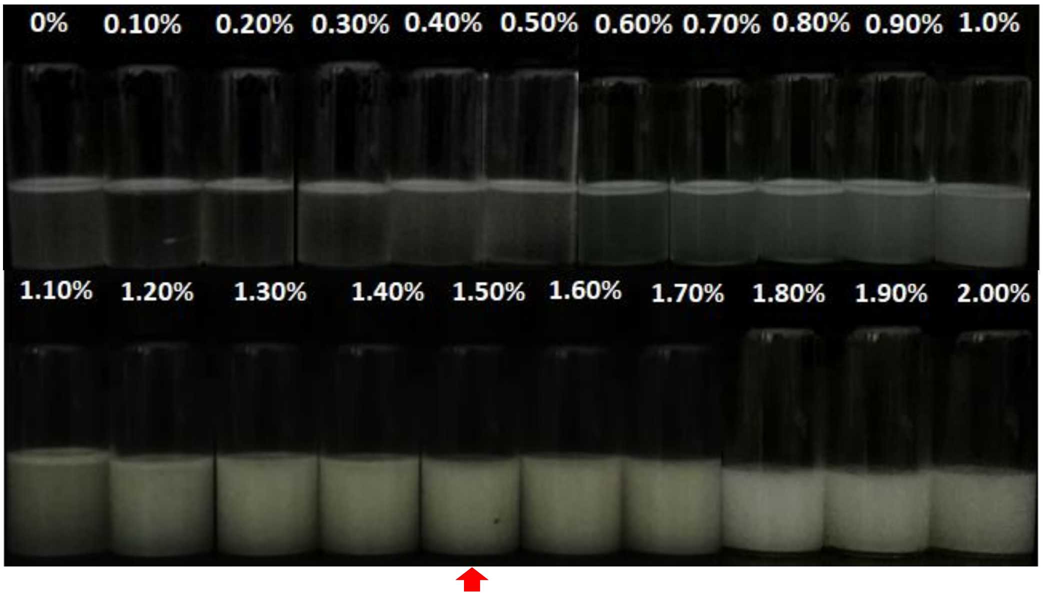

b

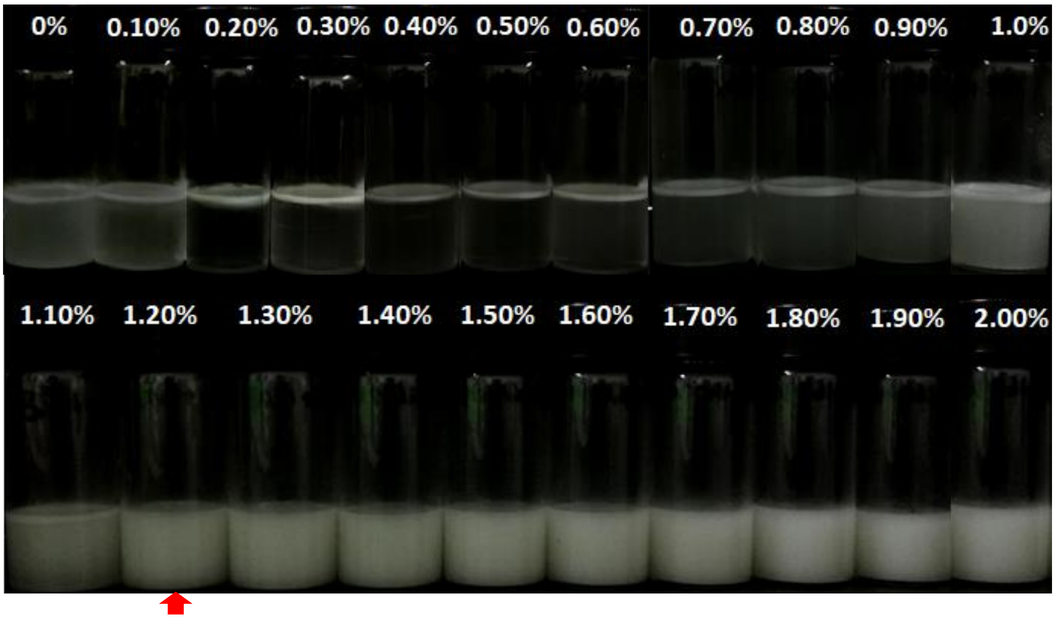

c

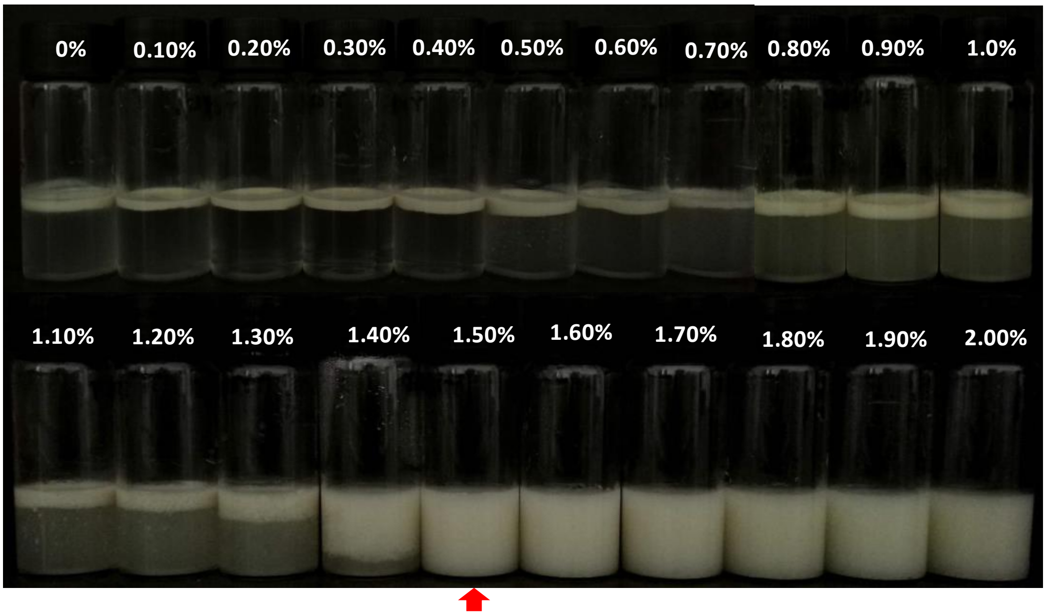

Figure S1: The creaming stability of (**a**) peanut, (**b**) sesame, and (**c**) rapeseed OB emulsions coated by different concentrations of ALG at pH 7. The OBs were dispersed in 50 mmol/L sodium phosphate buffer solution. The creaming observation was made after storage at 22 ± 2 ℃ for 7 days. The concentration shown on top of each sample represent the concentration of ALG.

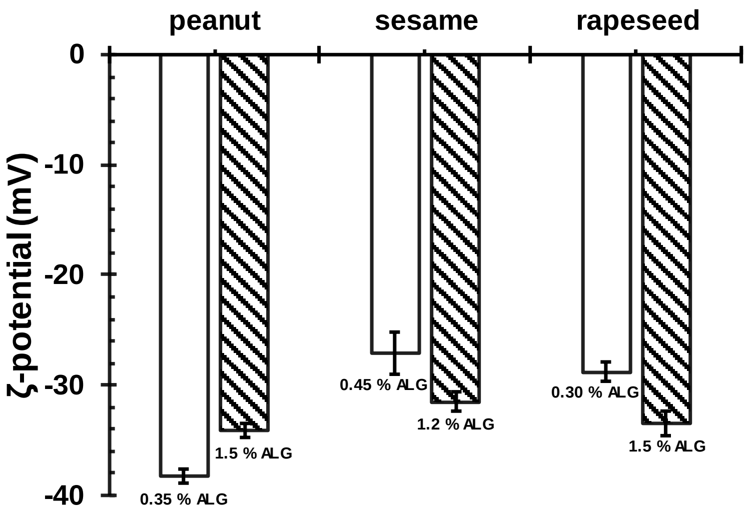

Figure S2: The ζ-potential of peanut, sesame, and rapeseed OBs (peanut, sesame, and rapeseed) stabilized by different concentrations of ALG at pH 7.

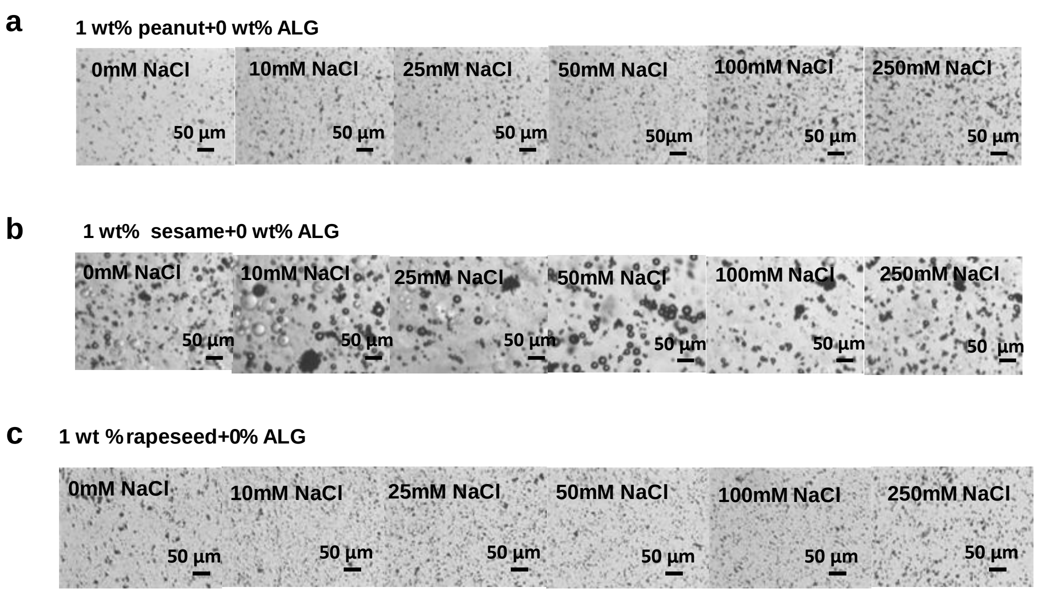

Figure S3: Salt effect on the microstructure of the OB emulsions (**a**) peanut, (**b**) sesame, and (**c**) rapeseed at pH 4. The OBs were dispersed in 50 mmol/L sodium phosphate buffer solution.

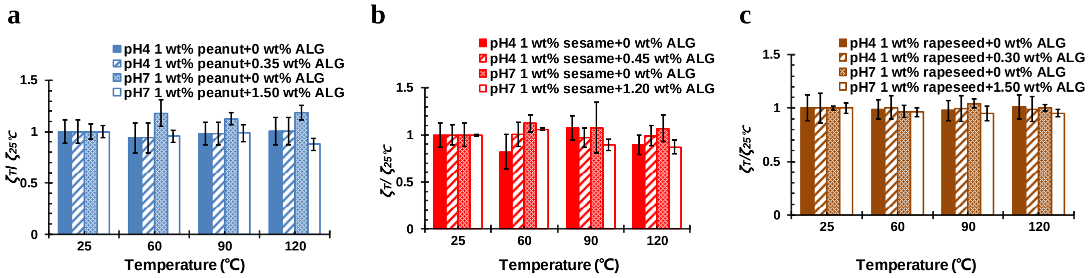

Figure S4: Effect of thermal treatment on relative ζ-potential ($\zeta_T/\zeta_{25℃}$) of pure and ALG stabilized OB emulsions at different pHs as indicated: (**a**) peanut, (**b**) sesame, and (**c**) rapeseed. The OBs were dispersed in 50 mmol/L PBS. ζ_T is the ζ-potential after heating at T ℃ for 30 min and $\zeta_{25℃}$ is the ζ-potential after heating at 25℃ for 30 min.

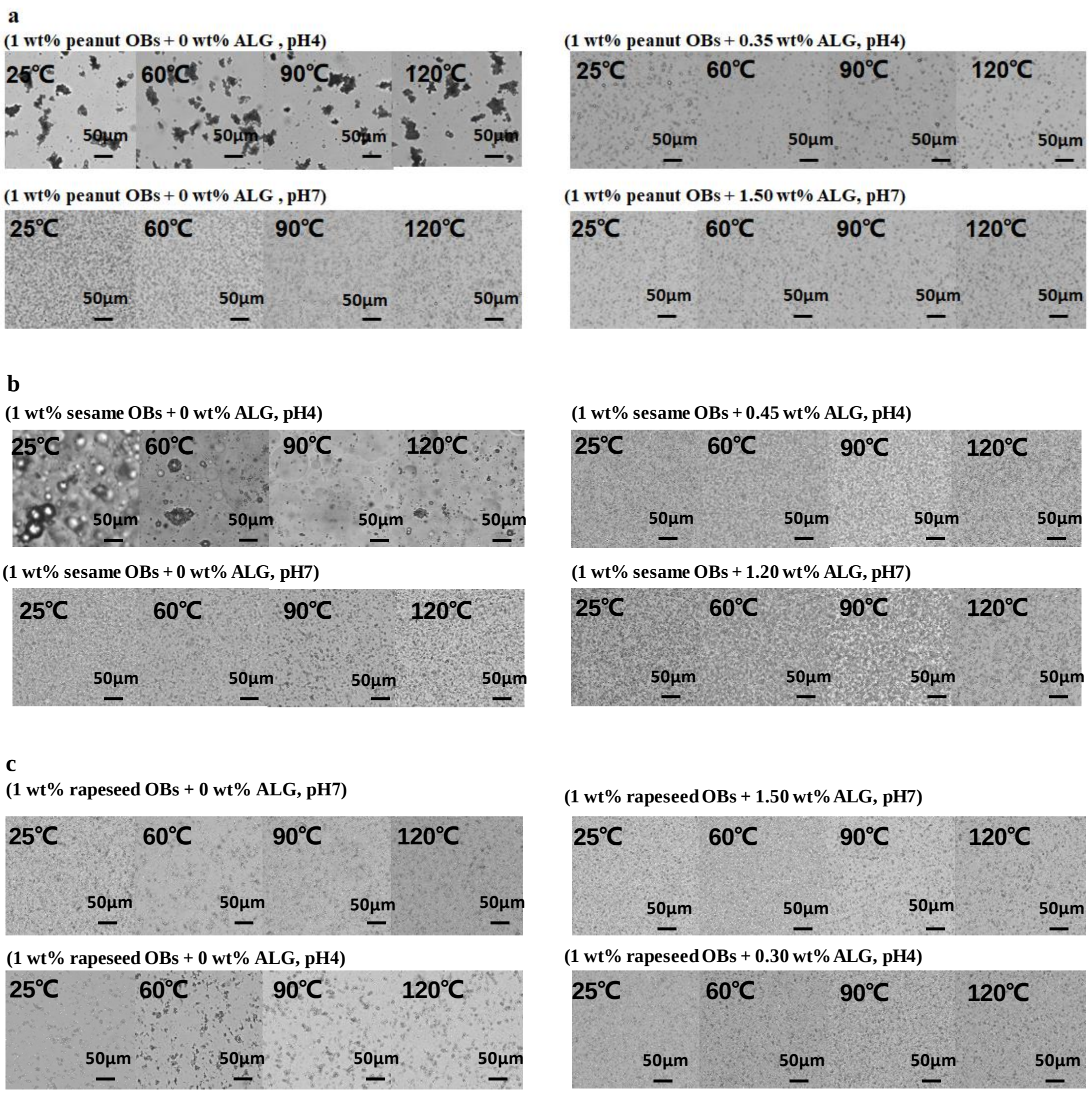

Figure S5: Effect of thermal treatment on the microstructure of pure and ALG stabilized OB emulsions at different pHs as indicated: (**a**) peanut, (**b**) sesame, and (**c**) rapeseed. The OBs were dispersed in 50 mmol/L PBS

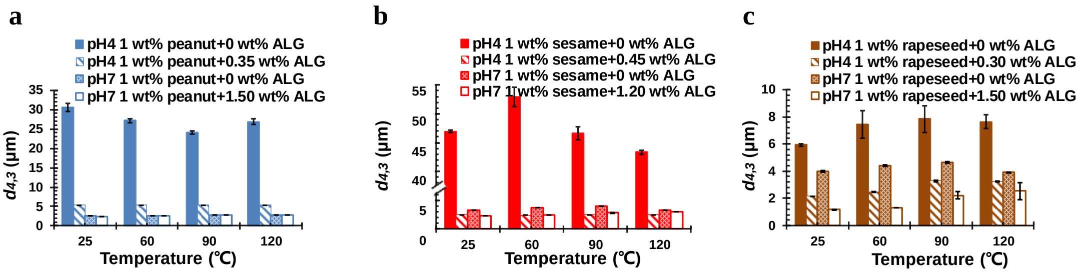

Figure S6: Effect of thermal treatment on mean particle diameter ($d_{4,3}$) of pure and ALG stabilized OB emulsions at different pHs as indicated: (**a**) peanut, (**b**) sesame, and (**c**) rapeseed. The OBs were dispersed in 50 mmol/L PBS
